# Strengthening laboratory capacity for detection of respiratory viral pathogens through the Global Health Security Agenda (GHSA) framework

**DOI:** 10.4102/ajlm.v8i1.861

**Published:** 2019-07-18

**Authors:** Brett Whitaker, Karen A. Alroy, Erica Guthrie, Sarah Schildecker, Susan Hiers, Jill Woodard, S. Arunmozhi Balajee

**Affiliations:** 1 Division of Viral Diseases, National Center for Immunization and Respiratory Diseases, Centers for Disease Control and Prevention, Atlanta, Georgia, United States; 2 Influenza Division, National Center for Immunization and Respiratory Diseases, Centers for Disease Control and Prevention, Atlanta, Georgia, United States; 3 Office of the Director, National Center for Immunization and Respiratory Diseases, Centers for Disease Control and Prevention, Atlanta, Georgia, United States

**Keywords:** public health laboratory, multiplex, respiratory viruses, global health security, polymerase chain reaction.

## Abstract

**Background:**

Endemic and emerging respiratory viruses are a threat to public health, and a robust public health laboratory system is essential to ensure global health security.

**Objective:**

This program sought to expand molecular laboratory testing capacity to detect a broad range of respiratory pathogens in clinical respiratory specimens collected during disease surveillance and outbreak investigations.

**Methods:**

As a part of the Global Health Security Agenda (GHSA), the United States Centers for Disease Control and Prevention utilised the equipment and training infrastructure already in place at the World Health Organization National Influenza Centers to expand testing capacity for respiratory viruses in laboratories in GHSA partner countries. This was done through the provision of quality assured reagents, including multiplex platforms and technical guidance for laboratory staff, as well as the assessment of laboratory testing accuracy.

**Conclusion:**

Early findings illustrated that GHSA laboratories have been able to expand testing capacity using specimens from routine surveillance, as well as from outbreak situations.

## Introduction

Acute respiratory infections have threatened public health for decades.^1,2,3,4^ In 2003, when severe acute respiratory syndrome emerged out of Hong Kong, it illustrated the speed with which respiratory pathogens could spread globally as well as their potential for worldwide economic impact.^5^ Two respiratory viruses have recently emerged as public health threats: influenza A/H7N9 and Middle East respiratory syndrome coronavirus.^6,7^ According to the International Health Regulations established by the World Health Organization (WHO), countries should develop capacities for timely detection and rapid public health response to emerging and re-emerging threats, and these capacities rely in part on a robust public health laboratory system.^8^

The Global Health Security Agenda (GHSA) (www.ghsagenda.org) is a partnership of countries and international organisations launched in 2014 to strengthen global capacity to detect, prevent and respond to disease threats that occur naturally, accidentally or deliberately. One of GHSA’s primary areas of focus, known as an ‘action package’, is an emphasis on strengthening national laboratory systems (Action Package: Detect 1).^9^ Through this action package, GHSA enables countries to enhance real-time bio-surveillance and ensure that laboratories are capable of safe, accurate detection and characterisation of epidemic-prone pathogens, including both known and novel threats.^9^ In order for public health laboratories to prepare for outbreaks and emerging pandemic threats, they should demonstrate ongoing capacities for routine diagnostics.

For almost a decade, the United States Centers for Disease Control and Prevention (CDC) Influenza Division has developed laboratory capacity for seasonal influenza virus and pandemic influenza preparedness. As a WHO collaborating center, CDC provides technical assistance for influenza testing in a network of global laboratories, known as National Influenza Centers (NICs), which have functional links to their assigned WHO reference laboratory and access to quality assured reagents from the CDC Influenza Division through the International Reagent Resource (IRR) (https://www.internationalreagentresource.org). The NICs often have standardised, state-of-the-art equipment, in addition to well-trained molecular biologists. Specimens for routine testing at NICs are received from influenza surveillance sites that include regional hospitals and clinics using the severe acute respiratory infection (SARI) or influenza-like illness case definitions. Frequently, SARI and influenza-like illness sentinel systems are models of successful surveillance in resource-limited countries where epidemiologic data are linked to specimen collection and laboratory testing.^10^ An opportunity exists to expand the existing surveillance systems at NICs in GHSA countries by expanding testing capacity for other priority agents.

Accordingly, as a part of the GHSA, the United States CDC strengthened and expanded its existing capacity to test for respiratory viral pathogens in laboratories of select countries (Table 1). These activities, detailed below, can serve as a model for other countries with NICs and laboratories with moderate technical capabilities that are looking to expand their capacities beyond influenza.

**TABLE 1:** Countries with expanded non-influenza virus reagent access that engaged in strengthening activities (February 2016–September 2017).

Country	No. of laboratories granted IRR access	No. of NIC laboratories	Total non-influenza respiratory virus products received from IRR	Participation date in basic training on respiratory viruses
Bangladesh	2	1	94	July 2016
Benin	0	0	0	April 2017
Burkina Faso	1	0	23	July 2016
Cameroon	1	1	37	July 2016
Côte D’Ivoire	1	1	28	July 2016
Democratic Republic of Congo	1	0	29	April 2017
Ethiopia	1	0	20	-
Gambia	1	0	30	-
Ghana	1	1	14	-
Guinea-Bissau	0	0	0	April 2017
India	2	1	20	-
Indonesia	1	1	4	-
Kenya	2	1	67	-
Mali	1	0	2	April 2017
Mauritania	1	0	7	April 2017
Pakistan	1	1	9	July 2016
Senegal	1	1	18	July 2016
Tanzania	1	1	14	July 2016
Togo	1	0	13	April 2017
Uganda	1	1	1	July 2016
Vietnam	4	2	72	July 2016

IRR, International Reagents Resource; NIC, National Influenza Center.

## Reagents for testing

In order to expand its laboratory capacity to test for respiratory viruses beyond influenza, CDC’s Division of Viral Diseases deployed a panel of in-house singleplex real-time reverse transcriptase polymerase chain reaction (rRT-PCR) assays (Table 2).^11,12,13,14,15^ Specifically, a subset of seven CDC in-house singleplex rRT-PCR assays and one specimen quality control assay RNase P (RNP) was distributed as a kit for SARI surveillance and outbreak investigations. All CDC in-house assays utilise Taqman^®^ probes (Applied Biosystems, Foster City, California, United States) with a 5’ FAM fluorophore and either an internally linked BHQ-1^®^ (Biosearch Technologies, Novato, California, United States) or 3’ terminal BHQ-1^®^ (Biosearch Technologies, Novato, California, United States) quencher. The RNP control allows laboratory scientists to know if the quality of nucleic acid has been compromised, potentially confounding any rRT-PCR results. These assays have been previously validated to work with several commercial one-step rRT-PCR enzyme kits such as the AgPath-ID^TM^ One-Step RT-PCR kit (Thermo Fisher, Waltham, Massachusetts, United States) and the qScript^®^ One-Step qRT-PCR kit, low ROX (Quantabio, Beverly, Massachusetts, United States). Real-time PCR instruments evaluated by CDC to be compatible with this reagent panel include the 7500 Fast Dx^TM^ (Applied Biosystems, Foster City, California, United States), Mx3000P^®^ and Mx3005P^®^ (Agilent, Santa Clara, California, United States), CFX96^TM^ and CFX96^TM^ Touch (Bio-Rad, Hercules, California, United States), and the Viia^TM^ 7 in both TaqMan^TM^ Array Card and 96-well PCR plate formats (Thermo Fisher, Waltham, Massachusetts, United States).^11,12,13,14,15,16^

**TABLE 2:** Human respiratory pathogens targeted by Centers for Disease Control and Prevention assays and fast-track diagnostics respiratory pathogens panel.

CDC non-influenza respiratory virus assays	FTD33 assays
Respiratory syncytial virus (RSV)†	Respiratory syncytial virus (HRSVA & B)
Human metapneumovirus (hMPV)†	Human metapneumovirus (HMPVA & B)
Human rhinovirus (hRV)†	Human rhinovirus (RV)
Human adenovirus (hAdv)†	Human adenovirus (HAdV)
Parainfluenza 1 (hPIV1)†	Human parainfluenza 1 (HPIV1)
Human parainfluenza 2 (hPIV2)†	Human parainfluenza 2 (HPIV2)
Human parainfluenza 3 (hPIV3)†	Human parainfluenza 3 (HPIV3)
RNase P (RNP)†	Human parainfluenza 4 (HPIV4)
Human parainfluenza 4 (hPIV4)	Influenza A, B, C, H1N1 (FLUA, FLUB, FLUC, H1N1)
Human coronaviruses 229E, OC43, NL63, HKU1	Human coronavirus (Cor63, Cor229, Cor43, HKU)
MERS-CoV assays upE, N2, and N3	Human bocavirus (HBoV)
	Enterovirus (EV)
	Human parechovirus (HPeV)
	*Mycoplasma pneumoniae* (Mpneu)
	*Staphylococcus aureus* (Saur)
	*Streptococcus pneumoniae* (Spneu)
	*Klebsiella pneumoniae* (Kpneu)
	*Moraxella catarrhalis* (Morax)
	*Haemophilus influenza/*b (Haeinf/Haeinfb)
	*Legionella* (Legio)
	*Pneumocystis jiroveci* (PCP)
	*Bordetella pertussis* (Bord)
	*Salmonella* species (Salm)
	*Chlamydia pneumoniae* (Cpneu)
	IC(EAV) – internal control

Note: Abbreviations in parentheses are shorthand assay names taken from the respective protocols/manuals for the pathogen targets listed.

CDC, Centers for Disease Control and Prevention; FTD, Fast-track Diagnostics.

† , Assays part of the CDC non-influenza respiratory virus real-time RT-PCR assay panel, for SARI surveillance/outbreaks.

When encountering an outbreak of unknown etiology, a multiplex platform may enable laboratories to rapidly identify the potential pathogen causing illness; therefore, CDC provided countries access to a multiplex assay kit known as FTD33 Respiratory Pathogens^®^ (Fast-track Diagnostics, Esch-sur-Alzette, Luxembourg), a commercially available multiplex assay that can detect up to 33 common respiratory viruses and bacteria. The FTD33 comes with its own proprietary primers, probes, PCR buffer, enzyme mix and controls needed to perform the tests. A previous version of the FTD33 kit, the FTD21 Respiratory Pathogens Kit, was validated against the CDC respiratory virus singleplex assay panel.^17^ Table 2 compares the pathogens targeted with the CDC seven-pathogen non-influenza respiratory virus panel and the FTD33.

### Ethical considerations

This article followed all ethical standards for a research without direct contact with human or animal subjects.

## Reagent procurement

Quality assured reagents are essential for accuracy in diagnostic testing. In addition, a reliable method for procurement and distribution of available reagents is necessary to order and receive laboratory materials, particularly when many of the reagents are temperature sensitive. Utilising GHSA funds, the online reagent portal, the IRR, was expanded to include 14 new custom products including the FTD33 kit, the CDC-developed respiratory virus rRT-PCR assay kits and controls, as well as ancillary commercial products such as rRT-PCR enzyme kits and specimen extraction kits required to run the CDC respiratory virus rRT-PCR assays. The IRR is a programme established by CDC to provide registered users with various reagents and information for the study and detection of influenza virus. It was chosen as the delivery mechanism for these reagents, because the selected laboratories for GHSA were already using or in the process of being approved to use the IRR for the purpose of WHO influenza surveillance, eliminating the need to establish a new or duplicative reagent distribution plan.

## Training workshops

In addition to enhancing laboratory capacity through the availability of quality reagents, CDC has enhanced its technical capacity through partnerships to conduct workforce training. Working with the Association of Public Health Laboratories, laboratory training took place in Atlanta in July of 2016. Nine countries participated and were trained in the expanded respiratory viral pathogen detection, including Bangladesh, Burkina Faso, Cameroon, Côte d’Ivoire, Pakistan, Senegal, Uganda, Tanzania and Vietnam. The Pasteur Institute of Paris, France, conducted similar laboratory training in Yaoundé, Cameroon, in April 2017 with representatives from Benin, Guinea-Bissau, Mali, Mauritania, the Democratic Republic of Congo and Togo. Table 1 lists countries that have received training up to December 2017.

The training curriculum included lectures from subject matter experts and hands-on laboratory exercises in order to familiarise attendees with the CDC rRT-PCR assays and the use of the FTD33. Didactic lectures covered a range of topics including an overview of respiratory viruses, quality assurance and biosafety guidelines, setting up a molecular diagnostics laboratory, and performing rRT-PCR assays on human clinical respiratory specimens. The hands-on exercises covered total nucleic acid isolation from clinical respiratory specimens, setting up rRT-PCR reactions for CDC singleplex and FTD33 multiplex assays, as well as analysis of the rRT-PCR data. Laboratory subject matter experts from CDC and the Association of Public Health Laboratories guided participants through the exercises ensuring everyone followed proper techniques. The specimens used for training were mock respiratory specimens spiked with inactivated respiratory viruses. By pre-characterising these specimens with the CDC respiratory virus panel, the participants’ results could be used as a metric for successful learning.

## Quality assessment

In order to test each country’s laboratory capabilities and availability of reagents following the training, CDC is preparing to provide laboratories with external quality assessment panels. These panels, produced by Quality Control for Molecular Diagnostics (http://www.qcmd.org), enable laboratories to process and test proficiency samples using the same protocol they use for clinical specimens. The results will be reported back to Quality Control for Molecular Diagnostics for scoring and the compilation of performance reports. Confidential individual reports will be returned to the respective laboratories. Based on their individual performances, CDC may recommend additional training and technical support, if necessary. For laboratories expanding their viral testing capacity, the availability of external quality assessment panels ensures quality performance.

## Preliminary summary data of expansion capacity

From February 2016 to September 2017, the IRR shipped 502 individual products, supporting respiratory virus rRT-PCR testing, to 25 different laboratories in 19 countries, and scientists from 15 countries received training (Table 1). Preliminary data from two of these laboratories indicated that a total of 362 respiratory specimens were tested, 158 by CDC singleplex respiratory virus assays and 204 by the FTD33 multiplex assay. It is hoped that in the future, the public health laboratory workforce in GHSA countries will collaborate with their epidemiology counterparts and utilise these reagents for the purposes of understanding the diversity of circulating respiratory viral pathogens, calculating the burden of disease for priority pathogens, and using multiplex platforms for outbreak detection.

### Next steps and conclusion

The GHSA measures its success according to metrics defined by the WHO’s Joint External Evaluation tool.^18^ In the arena of national laboratory systems, two of these indicators include the capacity for the detection of priority diseases, as well as for effective and modern diagnostics. The training and materials provided through CDC’s engagement in GHSA laboratory strengthening has provided tools and building blocks to more than one-third of all GHSA countries to more readily achieve their goals in laboratory capacity. Data generated from expanded testing will allow for a more complete understanding of the burden of disease in their country. A routine system of collection, transportation, testing and results sharing in a quality assured environment will enable the public health system to respond to outbreaks when they occur.

**Lessons learned**

Through the experiences described above, we identified key challenges and lessons learned where greater attention and investment may be warranted for future laboratory capacity building.

- The IRR is an important tool in the rapid expansion of testing capacities for the GHSA countries. Introducing new laboratory testing reagents through the IRR is a way of supporting countries that do not normally perform routine surveillance testing beyond influenza virus detection, and enables them to generate baseline data for additional pathogens. Centralising the distribution of custom CDC testing reagents along with commercial products through the IRR ensured that laboratories had a streamlined, single-stop source for vital, quality-assured laboratory reagents. This eliminates the need to get separate quotes for the same products specific to all the different laboratories. Additionally, by using the IRR as a reagent distribution mechanism, it is easy and fast to deploy new reagents for emergency situations, such as outbreaks of newly emergent disease.
- Developing and deploying training programmes with standardised curricula enables multiple laboratories and countries to practise uniform testing procedures, thereby allowing for better direct comparisons between countries and regions, and ultimately offering a better resolution in understanding the behaviour of respiratory viruses around the globe.
- In combination with strong epidemiological data, multiplex platforms such as FTD33 or other comparable products may provide valuable diagnostic information to better understand possible etiologies of respiratory disease, and ultimately improve detection capacities. Additionally, some multipathogen detection assays and platforms may still be cost prohibitive for many countries and public health systems, especially when used as a surveillance tool where the quantity of specimens needing testing can be quite high.
